# Higd1a Protects Cells from Lipotoxicity under High-Fat Exposure

**DOI:** 10.1155/2019/6051262

**Published:** 2019-04-08

**Authors:** Tong Li, Wen-jing Xian, Yang Gao, Shuai Jiang, Qi-hong Yu, Qi-chang Zheng, Yong Zhang

**Affiliations:** ^1^Department of Hepatobiliary Surgery, Union Hospital, Tongji Medical College, Huazhong University of Science and Technology, Wuhan 430022, China; ^2^Department of Anesthesiology, Union Hospital, Tongji Medical College, Huazhong University of Science and Technology, Wuhan 430022, China

## Abstract

Hypoxia-inducible gene domain family member 1A (Higd1a) has recently been reported to protect cells from hypoxia by helping to maintain normal mitochondrial function. The potential induction of Higd1a under high-fat exposure and whether it could protect cells from oxidative stress attracted our attention. Initially, 0.4 mM oleic acid and 0.2 mM palmitate were added to the growth media of HepG2 and LO2 cells for 72 hours. We discovered increased Higd1a expression, and knocking down Higd1a impaired mitochondrial transmembrane potential and induced cell apoptosis. We then identified that elevated reactive oxygen species (ROS) is responsible for increased Higd1a expression. Furthermore, we found that ROS promoted Higd1a expression by upregulating HIF-1a and PGC-1a expressions, and these two proteins could exert synergistic effects in inducing Higd1a expression. Taken together, these data suggest that Higd1a plays positive roles in protecting cells from oxidative stress, and ROS could induce Higd1a expression by upregulating PGC-1a and HIF-1a expressions.

## 1. Introduction

Nonalcoholic fatty liver disease (NAFLD) is one of the most prevalent liver diseases and is characterized by a wide range of alterations, including simple steatosis at early stages and steatohepatitis in advanced stages, in which fatty liver is accompanied by inflammation, hepatocyte ballooning, liver fibrosis, and disrupted glucose homeostasis and insulin resistance [1–3]. Due to the increasing prevalence of obesity and type II diabetes, the incidence of NAFLD is increasing dramatically, and NAFLD constitutes a global health concern, affecting not only adults but also children [4, 5]. Mitochondria play a central role in nutrient metabolism and provide energy required for a myriad of cell functions. In NAFLD patients, the rates of fatty acid oxidation (FAO) exceed the tricarboxylic acid cycle (TCA) capacity, resulting in mitochondrial fatty acid overload and leading to incomplete FAO and accumulation of reactive oxygen species (ROS) that contribute to mitochondria dysfunction and cell damage [6, 7]. Impaired mitochondrial *β*-oxidation and defective mitochondrial respiratory chain subsequently contribute to hepatic steatosis [8, 9]. Thus, protecting mitochondria from oxidative stress is one of the key aspects in treating NAFLD.

Hypoxia-induced gene domain protein-1a (Higd1a) (also known as HIG1 or HIMP1-a) is a 10.4 kDa mitochondrial inner membrane protein and is predicted to have two transmembrane domains oriented in an “N-terminal outside-C-terminal outside and loop inside” conformation [10, 11]. Higd1a is induced by hypoxia and ischemia and may aid in cell survival in the context of severe stress by protecting mitochondria [12–16]. Higd1a could also be induced during central nervous maturation. Under this circumstance, Higd1a may facilitate cell apoptosis and promote tissue remodeling [17]. Thus, the exact functions of Higd1a depend on the developmental stage and cellular microenvironment. In the present study, we found that Higd1a expression is increased under high-fat exposure and protects cells from oxidative stress. Moreover, we found that ROS promotes the expression of Higd1a by upregulating HIF-1a and PCG-1a. These results may help us to better understand the protective response of cells under oxidative stress.

## 2. Materials and Methods

### 2.1. Cells and Transfections

HepG2 and LO2 cells were purchased from the Cell Bank of Shanghai Institute of Cell Biology, Chinese Academy of Medical Sciences (Shanghai, China). These cells were maintained at 37°C in DMEM with 10% fetal bovine serum (Gibco, Invitrogen, USA). Higd1a siRNA (50 nM), HIF-1a siRNA (50 nM), PGC-1a siRNA (50 nM), pcDNA HIF-1a, pcDNA PGC-1a, and corresponding negative controls were designed and synthesized by RiboBio Co. (Guangzhou, China). Cell transfection was performed using Lipofectamine 3000 (Invitrogen, USA) according to the supplier's protocol. All sequences are provided in Supplementary Table 1.

### 2.2. Patients

Liver tissue specimens were obtained from patients who underwent liver resection for benign liver disease (polycystic liver or hepatic hemangioma), and the specimens were obtained at least 2 cm away from the liver lesion. NAFLD was identified by pathological examination. Specimens were stored in liquid nitrogen within 30 minutes after removal from patients during surgery. Informed consent forms were signed by all patients.

### 2.3. Animals

C57BL/6 mice were housed in sterilized isolators at 22 ± 1°C with 55-65% relative humidity and a 10 h light/14 h dark cycle. Mice were fed a standard diet (SD, composed of 20%, 70%, and 10% calories from protein, carbohydrate, and fat, respectively; Research Diets D12450B, Research Diets Inc., USA) or a high-fat diet (HFD, composed of 20%, 20%, and 60% calories from protein, carbohydrate, and fat, respectively; Research Diet D12492, Research Diets Inc., USA). Six weeks after feeding, adenovirus expressing Higd1a shRNA or control shRNA (Vigen Biosciences, Shandong, China) (the sequences are shown in Supplementary Table 1) at a titer of 4∗10^9^ was injected to the caudal vein of HFD-fed mice. All mice were sacrificed and analyzed 7 days after virus injection. The Animal Care and Use Committee of Union Hospital, Tongji Medical College, Huazhong University of Science and Technology, approved the animal protocol. The animal experiments adhered to the NIH *Guide for the Care and Use of Laboratory Animals*.

### 2.4. Preparation of FFAs

Sodium oleate and sodium palmitate were dissolved separately in ethanol at a concentration of 100 mM. Then, BSA solution (20%, *W*/*V*) was heated to 45°C, and oleate and palmitate solutions were added to the BSA solution to obtain a mixed FA stock solution (5 mM; oleate and palmitate at final ratio of 2 : 1). We also prepared single fatty acid stock solutions to which only oleate or palmitate solutions were added to the BSA solution. Stock solutions were then diluted in incubation medium containing 5 mg/ml of fatty acid-free BSA (Boehringer Mannheim GmbH, Mannheim, Germany) to desired concentrations. HepG2 and LO2 cells at 80% confluence were exposed to the FFAs for 72 hours.

### 2.5. Oil Red O Staining

Treated cells were washed three times with iced PBS and fixed with 4% paraformaldehyde for 30 minutes. After fixation, cells were washed three times and stained with Oil Red O solution (Shenggong, Shanghai, China) for 15 minutes at room temperature. Cells were then washed with PBS three times to remove redundant staining and observed under a microscope.

### 2.6. Inhibitor Studies

The antioxidant *N*-acetyl-L-cysteine was purchased from Sigma and was added to the growth media at a concentration of 1 mM.

### 2.7. Western Blotting and Antibodies

Total cell proteins were lysed in RIPA buffer with phosphatase inhibitors and a protease inhibitor cocktail (Sigma, USA). The following antibodies for western blotting were purchased from Santa Cruz (USA): mouse anti-Higd1a (1 : 1000), rabbit anti-HIF-1a, rabbit anti-PGC-1a (1 : 1000), and rabbit anti-GAPDH (1 : 1000). The rabbit/mouse secondary antibodies (1 : 3000) were obtained from Proteintech, China. Proteins were separated by 10% SDS-polyacrylamide gels and transferred to PVDF membranes. After blocking, blots were probed with specific antibodies at room temperature for 2 hours and then incubated with the appropriate secondary antibody. The protein bands were detected using a Bio-Rad imaging system (Bio-Rad, USA). All experiments were repeated at least three times, and the representative bands are indicated in the figures.

### 2.8. qRT-PCR

Total RNA was extracted from cells/liver tissues using TRIzol reagent (Invitrogen) according to the manufacturer's instructions. Reverse transcription was performed using a reverse transcription kit (Takara). Quantitative real-time reverse transcription PCR (qRT-PCR) was conducted using the quantitative SYBR Green PCR kit (Takara). Targets were normalized to glyceraldehyde-3-phosphate dehydrogenase (GAPDH). All qRT-PCR reactions were performed in triplicate. The qRT-PCR primers are presented in Supplementary Table 2.

### 2.9. Measurement of Mitochondrial Transmembrane Potential

Mitochondrial transmembrane potential (MMP) assay kit with JC-1 (Beyotime, China) was used to assess the MMP. Briefly, cells were incubated with JC-1 working fluid at 37°C for 20 minutes. After incubation, cells were washed twice with JC-1 dyeing buffer. Fluorescence was then detected using a fluorescence microscope (Olympus, Japan).

### 2.10. Measurement of Reactive Oxygen Species Production

After treatment of FFAs (or FFAs plus NAC) for 72 hours, 5 *μ*M DCFH-DA (2′,7′-dichlorodihydrofluorescein diacetate, Beyotime, China) was added to the cells and incubated for 30 minutes. Following the incubation period, the cells were washed with PBS and analyzed using flow cytometry.

### 2.11. Cell Proliferation Assay

A total of 5 × 10^3^ cells per well were seeded in 96-well plates. Seventy-two hours after FFA treatment, cell viability was determined using the CCK8 kit (Dojindo Laboratories, Japan) according to the manufacturer's protocol. The data were normalized to cells without drugs in each group.

### 2.12. Cell Apoptosis Assay

Cell apoptosis was assessed 72 hours after FFA treatment. Annexin V/propidium iodide staining was performed to quantify cell apoptosis. Cells were resuspended in 500 *μ*l of binding buffer, stained with Annexin V-FITC (5 *μ*l) and propidium iodide (10 *μ*l) in the dark for 15 minutes, and then analyzed using flow cytometry.

### 2.13. Statistical Analysis

Data are presented as the mean ± SE of three to four experiments. In appropriate cases, comparisons between groups were analyzed by ANOVA with post hoc analysis using Dunnett's multiple comparison tests. *P* values < 0.05 were considered statistically significant.

## 3. Results

### 3.1. Higd1a Expression Is Increased under High-Fat Exposure

**Figure 1:**
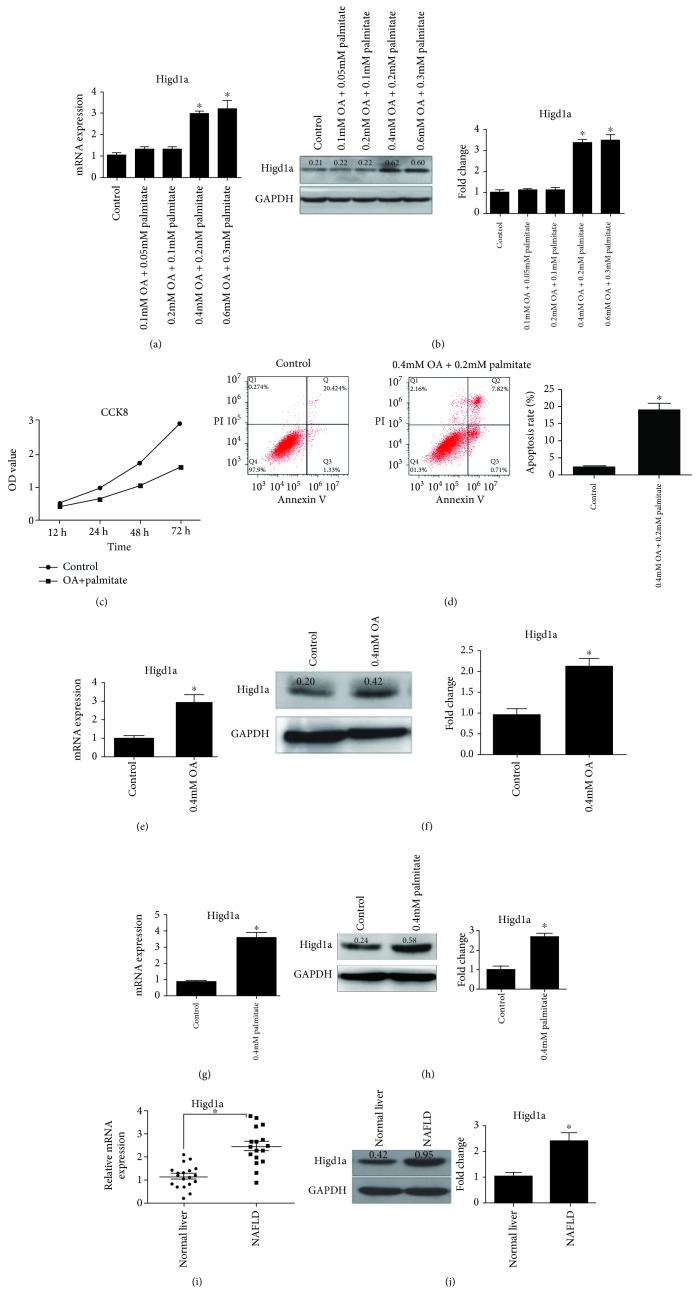
Higd1a expression is elevated under high-fat exposure. (a) LO2 cells were treated with various concentrations of oleic acid and palmitate (2 : 1) for 72 hours, and qRT-PCR and western blot (b) revealed that Higd1a expression was increased significantly upon exposure to 0.4 mM OA and 0.2 mM palmitate. (c) Cells were treated with 0.4 mM OA + 0.2 mM palmitate or untreated, and CCK8 assay revealed inhibition of cell proliferation in the FFA group. (d) Cell apoptosis was measured by flow cytometry using Annexin V/PI staining in the abovementioned groups, and more apoptosis was induced in the FFA group; LO2 cells were treated with 0.4 mM OA (e, f) or 0.4 mM palmitate (g, h) alone, and qRT-PCR and western blot were used to detect Higd1a expression in each group; Higd1a mRNA and protein expression in the livers of NAFLD patients was measured by qRT-PCR (i) and western blot (j) (^∗^ *P* < 0.05 compared with the control group).

Cells were treated with oleic acid (OA) and palmitate at different concentrations for 72 hours. Higd1a expression was significantly increased with 0.4 mM OA and 0.2 mM palmitate (Figures 1(a) and 1(b)). Oil Red staining revealed lipid droplets in the FFA group (0.4 mM OA + 0.2 mM palmitate) (Supplementary Figure 1). CCK8 assay and flow cytometry using Annexin V/PI staining were used to detect cell proliferation and apoptosis, respectively. Cell proliferation was inhibited in the FFA group, and more cell apoptosis had been induced (the apoptosis rate was 2.8 ± 1.2% in the control group versus 18.5 ± 4.5% in the FFA group) (Figures 1(c) and 1(d)). In addition, we found that OA (Figures 1(e) and 1(f)) or palmitate (Figures 1(g) and 1(h)) alone could also induce the expression of Higd1a. Moreover, the liver expression of Higd1a was elevated in NAFLD patients compared with those with a normal liver (Figures 1(i) and 1(j)). These results indicated that the expression of Higd1a could be induced under high-fat exposure.

### 3.2. Higd1a Protects Cells from Stress under High-Fat Exposure

**Figure 2:**
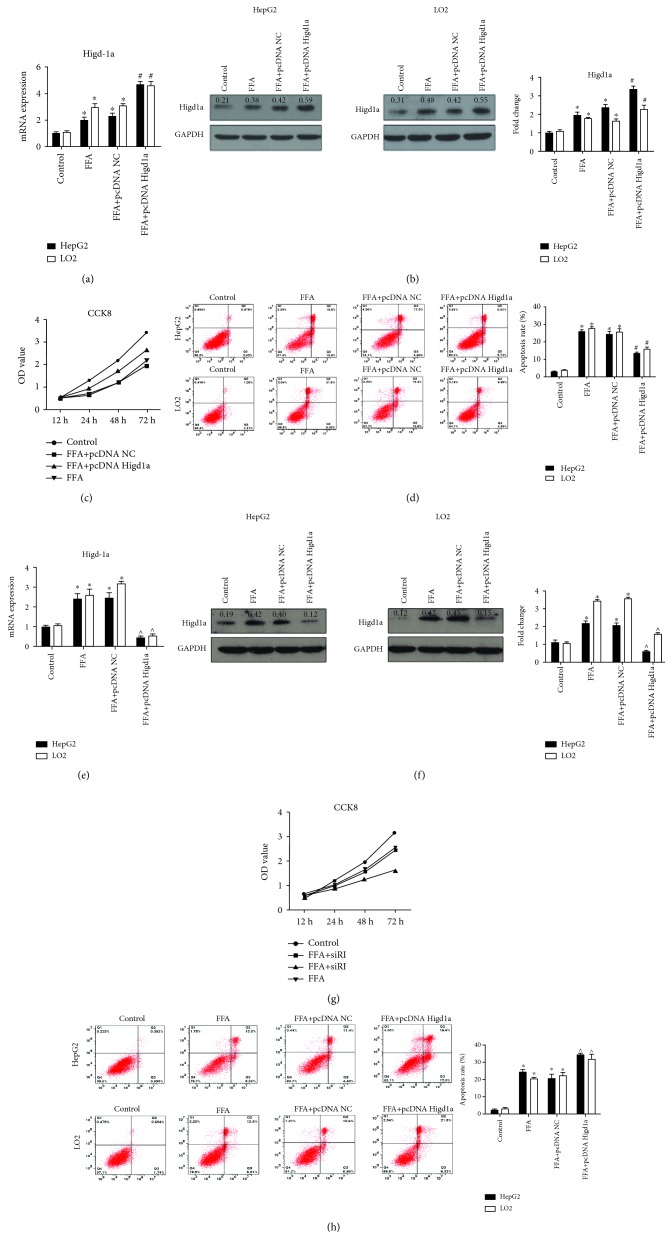
Overexpression of Higd1a protects cells from apoptosis under high-fat exposure and vice versa. (a) HepG2 and LO2 cells were transfected with pcDNA NC or pcDNA Higd1a and treated with FFAs (0.4 mM OA + 0.2 mM palmitate) for 72 hours, and the overexpression of Higd1a was validated by qRT-PCR and western blot (b); cells in the FFA+pcDNA Higd1a group exhibited increased expression compared with those in the FFA+pcDNA NC group. (c) Cell proliferation in the abovementioned groups was measured by the CCK8 kit. (d) Cell apoptosis was assessed by flow cytometry using Annexin V/PI staining, and less apoptosis was induced in the FFA+pcDNA Higd1a group compared with the FFA+pcDNA NC group. (e) Cells were transfected with siRNA NC or siRNA Higd1a and then treated with FFAs for 72 hours; Higd1a knockdown was validated by qRT-PCR and western blot (f). (g) Cell proliferation in each group was measured by the CCK8 kit, and cell proliferation was more inhibited in the FFA+siRNA Higd1a group compared with the FFA+siRNA NC group. (h) Cell apoptosis in each group was measured by flow cytometry using Annexin V/PI staining, and more apoptosis was induced in the FFA+siRNA Higd1a group compared with the FFA+siRNA NC group (^∗^ *P* < 0.05 compared with the control group; ^#^ *P* < 0.05 compared with the FFA+pcDNA NC group; ^∧^ *P* < 0.05 compared with the FFA+siRNA NC group).

**Figure 3:**
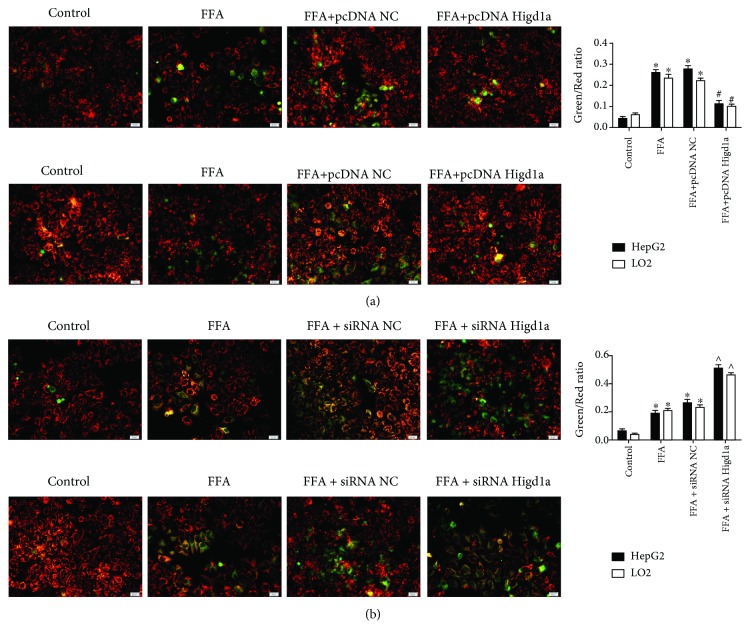
Overexpression of Higd1a protects mitochondrial transmembrane potential under high-fat exposure and vice versa. (a) Mitochondrial transmembrane potential (MMP) in the control group, FFA group, FFA+pcDNA NC group, and FFA+pcDNA Higd1a group was measured by fluorescence microscopy (Olympus, Japan) (JC-1 dye was used to evaluate MMP; red fluorescence indicated cells with normal MMP, and green fluorescence indicated cells with impaired MMP), and the impairment of MMP was less severe in the FFA+pcDNA Higd1a group compared with the FFA+pcDNA NC group. (b) MMP in the control group, FFA group, FFA+siRNA NC group, and FFA+siRNA Higd1a group was measured by fluorescence microscopy, and the impairment of MMP was more severe in the FFA+siRNA Higd1a group compared with the FFA+siRNA NC group (^∗^ *P* < 0.05 compared with the control group; ^#^ *P* < 0.05 compared with the FFA+pcDNA NC group; ^∧^ *P* < 0.05 compared with the FFA+siRNA NC group).

Given that previous studies demonstrated the protective effects of Higd1a on cells under hypoxic conditions (12), we then detected whether it could protect cells from stress under high-fat exposure. HepG2 and LO2 cells were transfected with pcDNA NC or pcDNA Higd1a and treated with FFAs for 72 hours. Higd1a overexpression was confirmed by qRT-PCR and western blot (Figures 2(a) and 2(b)). CCK8 assay and flow cytometry using Annexin V/PI staining revealed increased cell proliferation and decreased cell apoptosis in the FFA+pcDNA Higd1a group compared with the FFA+pcDNA NC group (apoptosis rate in HepG2/LO2 cells was 13.33 ± 1.53%/16.00 ± 1.63% in the FFA+pcDNA Higd1a group versus 24.33 ± 3.06%/25.67 ± 2.49% in the FFA+pcDNA NC group) (Figures 2(c) and 2(d)). Cells were then transfected with siRNA NC or siRNA Higd1a and treated with FFAs; Higd1a knockdown was validated by qRT-PCR and western blot (Figures 2(e) and 2(f)). As expected, cell proliferation was further inhibited in the FFA+siRNA Higd1a group, and more apoptosis was induced in the FFA+siRNA Higd1a group compared with the FFA+siRNA NC group (apoptosis rate in HepG2/LO2 cells was 33.67 ± 2.08%/31.67 ± 3.40% in the FFA+siRNA Higd1a group versus 20.67 ± 4.16%/21.67 ± 2.87% in the FFA+siRNA NC group) (Figures 2(g) and 2(h)). Given that Higd1a is localized to the mitochondrial inner membrane, we detected the impact of Higd1a on mitochondrial transmembrane potential (MMP). As expected, upregulating Higd1a before treatment with FFAs protected MMP, whereas downregulating Higd1a exerted opposite effects (Figures 3(a) and 3(b)). These results suggest that Higd1a plays positive roles in protecting cells from stress under high-fat exposure.

### 3.3. ROS Increases Higd1a Expression under High-Fat Exposure

**Figure 4:**
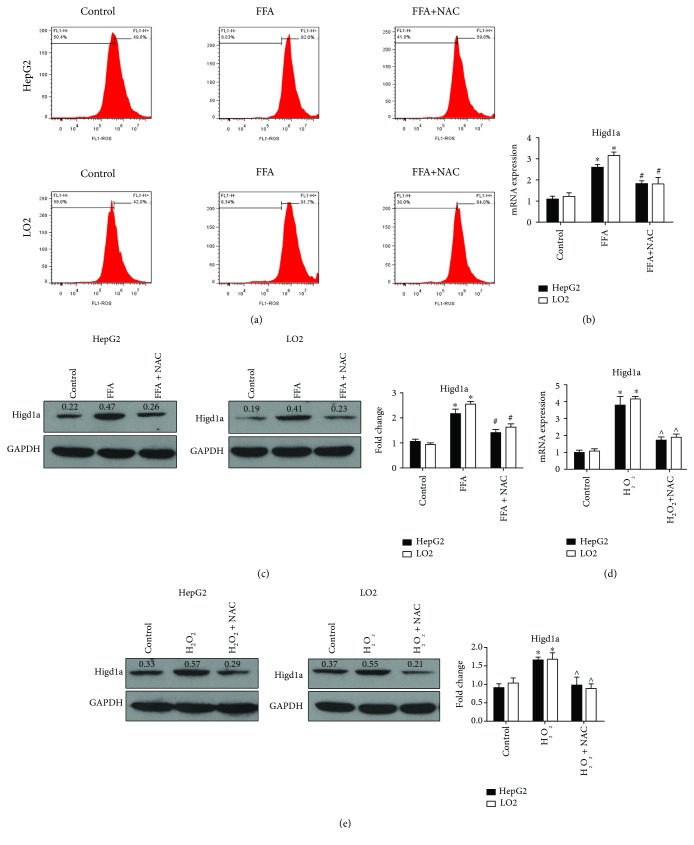
ROS promote Higd1a expression under high-fat exposure. (a) Flow cytometry using DCFH-DA identified intracellular ROS production in the control group, FFA group, and FFA+NAC group, and intracellular ROS was significantly elevated in the FFA group. (b) qRT-PCR and western blot (c) detected Higd1a expression in the abovementioned groups, and Higd1a expression was elevated in the FFA group but decreased in the FFA+NAC group. (d) qRT-PCR and western blot (e) detected the expression of Higd1a in the control group, H_2_O_2_ group, and the H_2_O_2_+NAC group, and Higd1a expression was increased in the H_2_O_2_ group but decreased in the H_2_O_2_+NAC group (^∗^ *P* < 0.05 compared with the control group; ^#^ *P* < 0.05 compared with the FFA group; ^∧^ *P* < 0.05 compared with the H_2_O_2_ group).

The production of ROS is accelerated under lipotoxicity, and ROS induce a large series of proteins related to energy metabolism [18]. We then investigated whether elevated ROS could induce Higd1a expression. Flow cytometry using DCFH-DA staining revealed elevated intracellular ROS in the FFA group (cells treated with 0.4 mM OA + 0.2 mM palmitate) (Figure 4(a)). We then added the ROS inhibitor *N*-acetyl-L-cysteine (NAC) to the FFA group and assessed Higd1a expression. Interestingly, both Higd1a mRNA and protein levels were decreased (Figures 4(b) and 4(c)). In addition, Higd1a expression increased when cells were treated with hydrogen peroxide (H_2_O_2_), the established ROS inducer, and the expression decreased after cells were treated with NAC (Figures 4(d) and 4(e)). These results indicate that ROS could induce the expression of Higd1a under high-fat exposure.

### 3.4. ROS Promote the Expression of Higd1a by Upregulating HIF-1a under High-Fat Exposure

**Figure 5:**
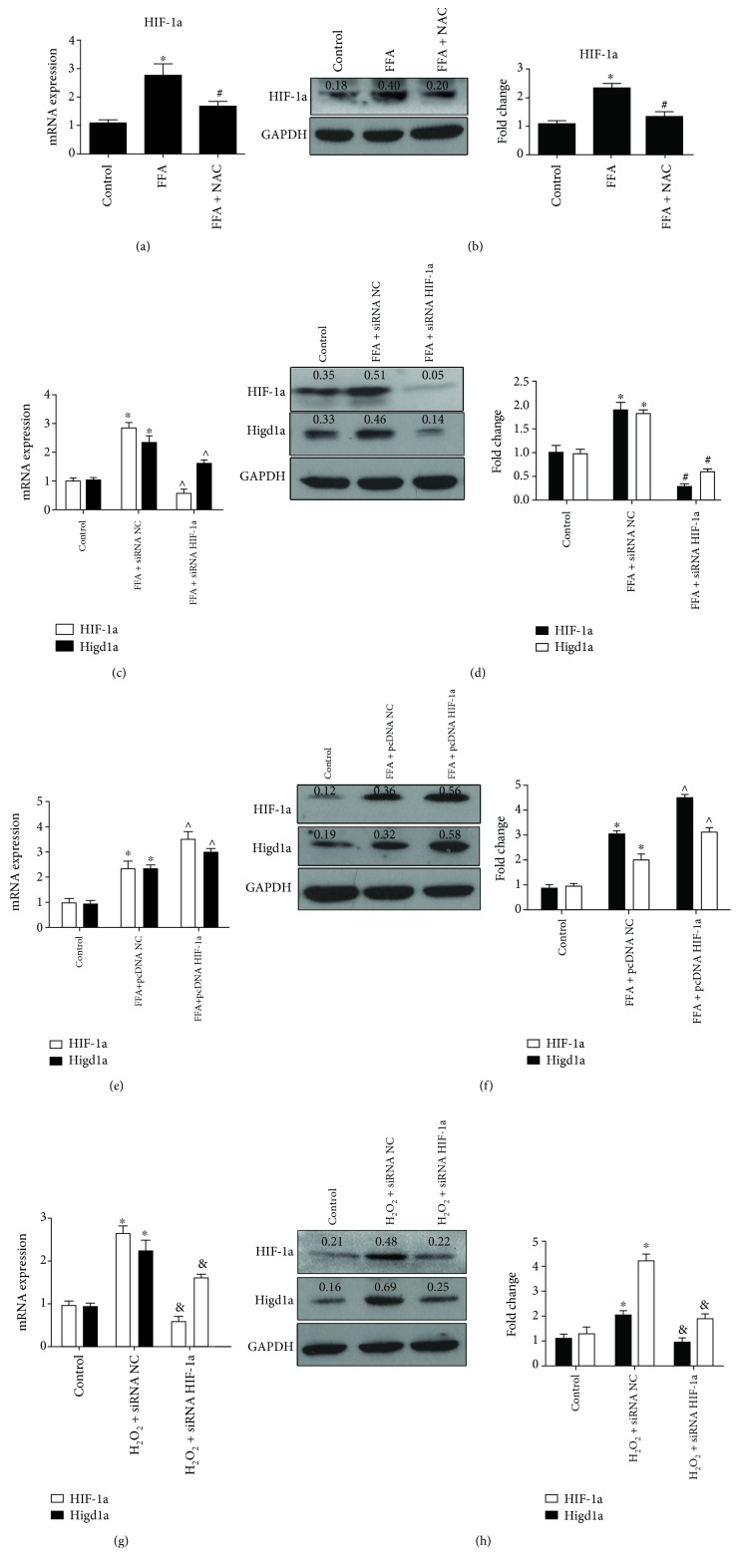
ROS promote Higd1a expression by upregulating HIF-1a. (a) HIF-1a expression in the control group, FFA group, and FFA+NAC group was measured by qRT-PCR and western blot (b), and HIF-1a expression was elevated in the FFA group but decreased in the FFA+NAC group. (c) Cells were transfected with siRNA NC or siRNA HIF-1a and then treated with FFAs; qRT-PCR and western blot (d) were used to detect Higd1a expression in each group, and Higd1a expression was decreased in the FFA+siRNA HIF-1a group compared with the FFA+siRNA NC group. (e) Cells were transfected with pcDNA NC or pcDNA HIF-1a and then treated with FFAs, and qRT-PCR and western blot (f) demonstrated that Higd1a expression was increased in the FFA+pcDNA HIF-1a group compared with the FFA+pcDNA NC group. (g) Cells were transfected with siRNA NC or siRNA HIF-1a and then treated with H_2_O_2_, and qRT-PCR and western blot (h) revealed that HIF-1a and Higd1a expressions were increased in the H_2_O_2_+siRNA NC group but decreased in the H_2_O_2_+siRNA HIF-1a group (^∗^ *P* < 0.05 compared with the control group; ^#^ *P* < 0.05 compared with the FFA group; ^∧^ *P* < 0.05 compared with the FFA+siRNA NC (pcDNA NC) group; ^&^ *P* < 0.05 compared with the H_2_O_2_+siRNA HIF-1a group).

HIF-1a is a master transcriptional regulator of the cellular response to hypoxia and induces the transcription of genes involved in cell proliferation, cell survival, and cell metabolism [19]. Previous studies reported that Higd1a may be induced by HIF-1a under hypoxic conditions, but different points of views exist [12, 13]. Given that HIF-1a may also be induced by ROS, we then investigated whether HIF-1a played a role in ROS-mediated Higd1a expression. As expected, HIF-1a expression was increased in the FFA group and returned to approximately normal levels when NAC was added to the culture media (Figures 5(a) and 5(b)). Cells were then transfected with siRNA NC, siRNA HIF-1a, pcDNA NC, or pcDNA HIF-1a, separately, and treated with FFAs for 72 hours. Interestingly, Higd1a expression in the FFA+siRNA HIF-1a group was considerably reduced compared with that in the FFA+siRNA NC group (Figures 5(c) and 5(d)). In contrast, Higd1a expression was considerably increased in the FFA+pcDNA HIF-1a group compared with the FFA+pcDNA NC group (Figures 5(e) and 5(f)). Moreover, cells transfected with siRNA HIF-1a exhibited reduced Higd1a expression compared with cells transfected with siRNA NC when treated with H_2_O_2_ (Figures 5(g) and 5(h)). These results suggest that ROS promote Higd1a expression partially through upregulating HIF-1a.

### 3.5. ROS Promote Higd1a Expression by Upregulating PGC-1a under High-Fat Exposure

**Figure 6:**
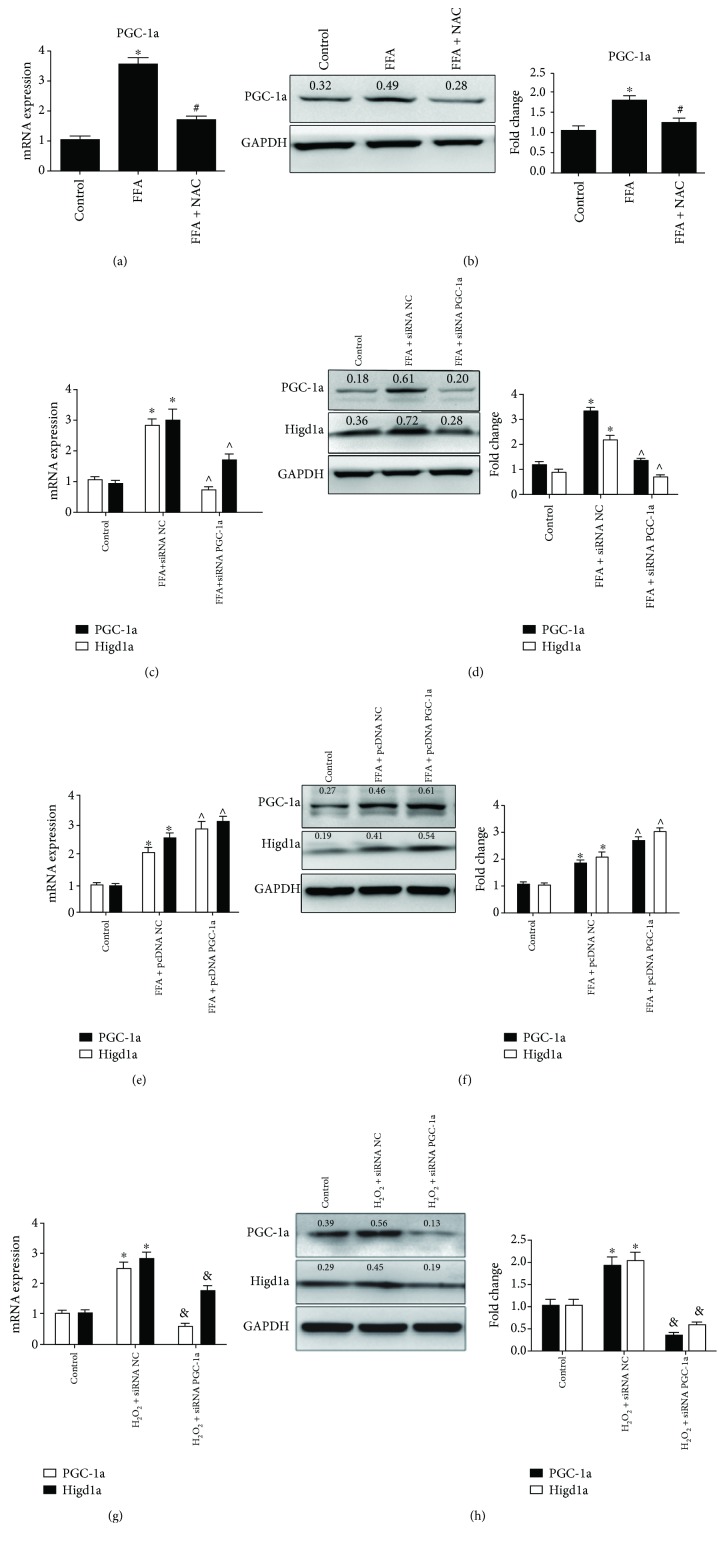
ROS promote Higd1a expression by upregulating PGC-1a. (a) PGC-1a expression in the control group, FFA group, and FFA+NAC group was measured by qRT-PCR and western blot (b), and levels were elevated in the FFA group but decreased in the FFA+NAC group. (c) Cells were transfected with siRNA NC or siRNA PGC-1a and then treated with FFAs; qRT-PCR and western blot (d) were used to detect Higd1a expression in each group, and Higd1a expression was decreased in the FFA+siRNA PGC-1a group compared with the FFA+siRNA NC group. (e) Cells were transfected with pcDNA NC or pcDNA PGC-1a and then treated with FFAs, and qRT-PCR and western blot (f) demonstrated that Higd1a expression was increased in the FFA+pcDNA PGC-1a group compared with the FFA+pcDNA NC group. (g) Cells were transfected with siRNA NC or siRNA PGC-1a and then treated with H_2_O_2_, and qRT-PCR and western blot (h) demonstrated that PGC-1a and Higd1a expressions were increased in the H_2_O_2_+siRNA NC group but decreased in the H_2_O_2_+siRNA PGC-1a group (^∗^ *P* < 0.05 compared with the control group; ^#^ *P* < 0.05 compared with the FFA group; ^∧^ *P* < 0.05 compared with the FFA+siRNA NC (pcDNA NC) group; ^&^ *P* < 0.05 compared with the H_2_O_2_+siRNA PGC-1a group).

PGC-1a is a powerful transcriptional coactivator that regulates cell metabolism and mitochondrial biogenesis. Sen et al. [20] reported that PGC-1a acts as a key modulator of P53 and promotes cell survival upon metabolic stress. PGC-1a is also induced by ROS under oxidative stress [21], so we investigated whether PGC-1a regulates the expression of Higd1a under high-fat exposure. First, we found that PGC-1a expression was significantly increased in the FFA group, whereas minimal changes in expression were noted in the FFA plus NAC group (Figures 6(a) and 6(b)). We then transfected cells with siRNA NC, siRNA PGC-1a, pcDNA NC, or pcDNA PGC-1a, separately, and treated cells with FFAs. We found that Higd1a mRNA and protein expression were decreased if PGC-1a was knocked down before cells were treated with FFAs (Figures 6(c) and 6(d)), whereas expression was increased if PGC-1a was overexpressed (Figures 6(e) and 6(f)). In addition, knocking down PGC-1a before cells were treated with H_2_O_2_ reduced Higd1a expression (Figures 6(g) and 6(h)). These results indicated that PGC-1a is involved in ROS-induced upregulation of Higd1a.

### 3.6. PGC-1a and HIF-1a Exert a Synergistic Effect on Inducing Higd1a Expression

**Figure 7:**
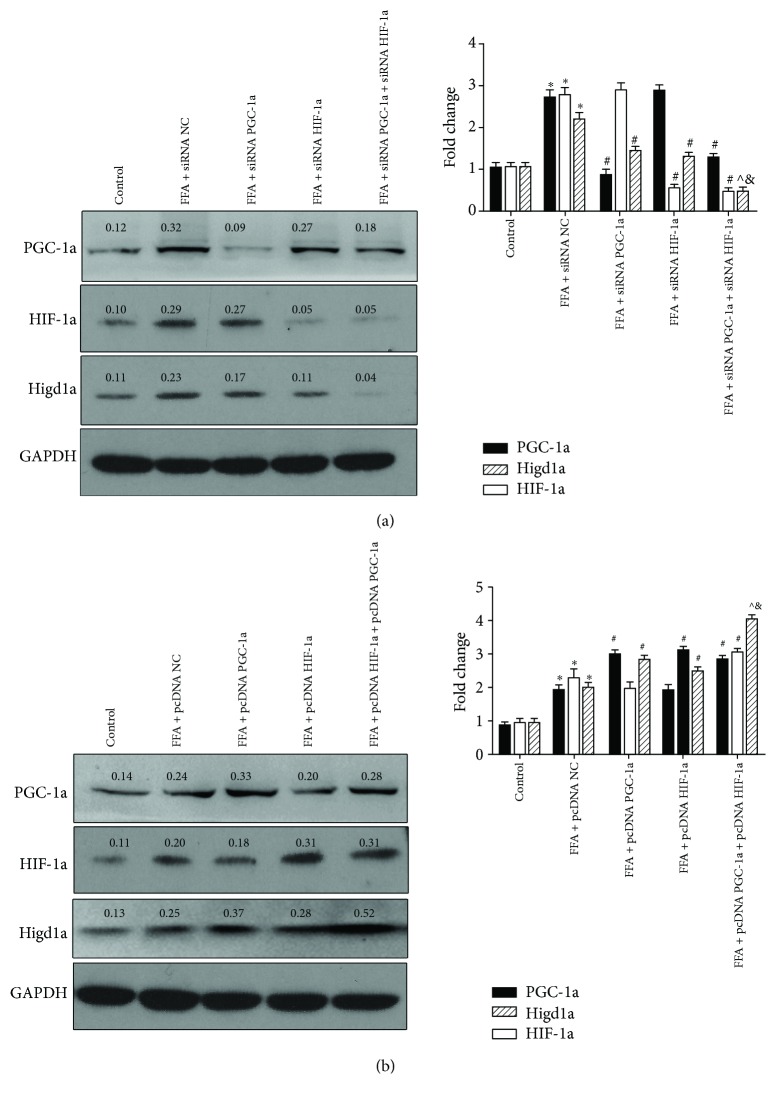
PGC-1a and HIF-1a exert synergistic effects on inducing Higd1a expression. (a) LO2 cells were transfected with siRNA NC, siRNA PGC-1a, siRNA HIF-1a, or siRNA PGC-1a+siRNA HIF-1a and then treated with FFAs; the protein expression of PGC-1a, HIF-1a, and Higd1a was measured by western blot, and Higd1a expression was decreased in the FFA+siRNA PGC-1a+siRNA HIF-1a group compared with other groups. (b) LO2 cells were transfected with pcDNA NC, pcDNA PGC-1a, pcDNA HIF-1a, or pcDNA PGC-1a+pcDNA HIF-1a and then treated with FFAs, and western blot revealed that Higd1a expression was increased in the FFA+pcDNA PGC-1a+pcDNA HIF-1a group compared with other groups (^∗^ *P* < 0.05 compared with the control group; ^#^ *P* < 0.05 compared with FFA+siRNA NC group or FFA+pcDNA NC group; ^∧^ *P* < 0.05 compared with the FFA+siRNA PGC-1a group or FFA+pcDNA PGC-1a group; ^&^ *P* < 0.05 compared with the FFA+siRNA HIF-1a group or FFA+pcDNA HIF-1a group).

Given that both PGC-1a and HIF-1a exhibited regulatory effects on Higd1a, we then investigated the synergistic role of these two genes in regulating Higd1a. Cells were transfected with siRNA NC, siRNA PGC-1a, siRNA HIF-1a, or siRNA PGC-1a plus HIF-1a, separately. FFAs were added to the culture medium after transfection, and Higd1a protein expression was detected by western blot. As expected, the group cotransfected with siRNA PGC-1a and HIF-1a exhibited reduced Higd1a expression compared with other groups (Figure 7(a)). Next, we transfected cells with pcDNA NC, pcDNA PGC-1a, pcDNA HIF-1a, or pcDNA PGC-1a plus pcDNA HIF-1a and treated cells with FFAs. Western blot revealed increased Higd1a expression in the FFA+pcDNA PGC-1a+pcDNA HIF-1a group compared with other groups (Figure 7(b)). These results suggest that PGC-1a and HIF-1a exert a synergistic effect in inducing Higd1a expression.

### 3.7. Knocking Down Higd1a Aggravated Liver Injury in NAFLD Mice

**Figure 8:**
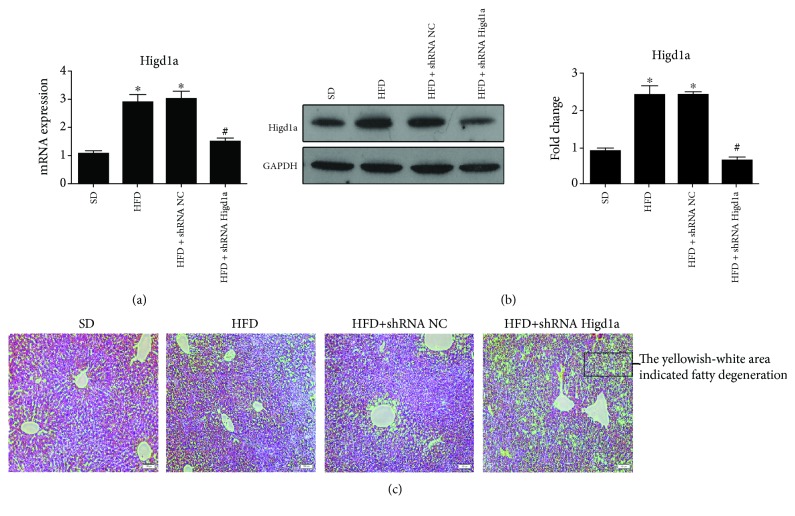
Knocking down Higd1a aggravated liver injury in NAFLD mice. Mice were fed a standard diet (SD group) or high-fat diet (HFD group). Mice fed a HFD were subjected to caudal vein injection with adenoviral virus expressing Higd1a shRNA (HFD+shRNA Higd1a group) or controlled shRNA (HFD+shRNA NC group), and qRT-PCR (a) and western blot (b) were used to measure Higd1a mRNA and protein expression in each group; liver tissues in each group were stained with HE for histological examination, and more severe liver injury was noted in the HFD+shRNA Higd1a group compared with other groups (c) (^∗^ *P* < 0.05 compared with the SD group; ^#^ *P* < 0.05 compared with the HFD+shRNA NC group).

We established an in vitro model to demonstrate that increased Higd1a expression exerted a positive role in protecting cells from oxidative stress, and we next explored whether Higd1a had similar in vivo effects. Mice were fed a standard diet (SD) or a high-fat diet (HFD) for 6 weeks. Mice fed a HFD were then subjected to caudal vein injection adenoviral virus expressing a shRNA against Higd1a or a control shRNA at a titer of 4∗10^9^. After 7 additional days, all mice were sacrificed and analyzed. As expected, Higd1a expression in the liver was increased in mice fed a HFD compared with mice fed a SD and reduced in mice injected with adenovirus expressing Higd1a shRNA (Figures 8(a) and 8(b)). Liver tissues were then stained with HE for histological examination. Mice fed a HFD developed a fatty liver, and hepatic steatosis was more severe in mice injected with adenovirus expressing Higd1a shRNA (Figure 8(c)). These results enhanced the positive role of Higd1a in protecting cells from oxidative stress.

## 4. Discussion

Based on our research, this is the first study to demonstrate that Higd1a is elevated under high-fat exposure, potentially protecting cells from oxidative stress. We first demonstrated that ROS promote the expression of Higd1a by inducing HIF-1a and PGC-1a.

Hypoxia-inducible gene domain family member 1A (Higd1a) is a HIF-1a targeted mitochondrial protein that is typically induced by hypoxia. Higd1a binds to mitochondrial *γ*-secretase complex, decreases *γ*-secretase activity, and thus reduces ROS production and mitochondrial dysfunction [14]. Higd1a also interacts with the mitochondrial electron transport chain and decreases oxygen consumption and AMPK activity to promote cell survival [11]. Moreover, Higd1a binds to the cytochrome c oxidase complex and increases its activity, thus protecting cells from an energy crisis [15]. In this study, Higd1a expression was elevated under high-fat exposure. In addition, knocking down Higd1a impaired MMP and induced cell apoptosis and vice versa. These results indicate that Higd1a plays a positive role in protecting cells from oxidative stress under high-fat exposure, and Higd1a may represent a potential target in treating NAFLD. Higd1a is localized to the mitochondria, and a prior report suggests that Higd1a is translocated from the mitochondria to the nucleus upon metabolic stress [22]. Similar to AIF, GAPDH, or FKP51, Higd1a may also exhibit novel nuclear roles that could fine tune cellular fates [23–25]. Although we did not explore the exact mechanism by which Higd1a protects cells, we hypothesize that some key proteins related to cell death or survival may be regulated by Higd1a, and this hypothesis will be further assessed in future research.

Whether HIF-1a promotes Higd1a expression is controversial. An et al. [13] reported that HIF-1a could bind to the promoter of Higd1a and induce Higd1a expression. However, Ameri and Maltepe [12] reported that Higd1a is not induced by HIF-1a in hypoxic cells but is instead triggered by additional metabolic stressors. If the promoter of Higd1a is methylated, its expression is inhibited. In this study, we discovered that HIF-1a expression is elevated in the fatty acid group. Knocking down HIF-1a reduced the expression of Higd1a and vice versa. These results indicate that HIF-1a induces Higd1a expression under high-fat exposure.

PGC-1a is a transcriptional coactivator that regulates genes involved in energy metabolism and exhibits powerful transcriptional activity when linked to a heterologous DNA-binding domain or docked to a transcription factor [26]. Under stress, PGC-1a activates gluconeogenesis, fatty acid *β*-oxidation, ketogenesis, and bile acid homeostasis by coactivating key hepatic transcription factors, such as HNF4a, FOXO1, GR, and FXR [27–31]. Loss of PGC-1a leads to significant functional deficits in oxidative metabolism and exacerbates steatohepatitis. This is the first study to identify that together with HIF-1a, PGC-1a also promotes Higd1a expression under high-fat exposure, and these results could help us to better understand Higd1a regulation.

When cells are exposed to high levels of fat, *β*-oxidation is accelerated. In addition, more electrons may leak from the respiratory chain, and more ROS will be generated [32]. Our study first demonstrated that elevated ROS could promote the expression of Higd1a potentially by upregulating HIF-1a and PGC-1a. Although our research is preliminary, these results could promote more studies in this field.

In conclusion, our study found that increased Higd1a expression could protect cells from oxidative stress, and ROS induce Higd1a expression by upregulating HIF-1a and PGC-1a expressions upon high-fat exposure.

## Data Availability

The data used to support the findings of this study are available from the corresponding author upon request.

## Supplementary Materials

Supplementary Materials Supplementary Figure 1: Oil Red staining identified lipid droplets in the FFA (0.4 mM OA + 0.2 mM palmitate) group. Supplementary Table 1: sequences of siRNA. Supplementary Table 2: primer sequences for qRT-PCR used in the study.
