# Supplementary material for: Higd1a Protects Cells from Lipotoxicity under High-Fat Exposure
Source: Oxid Med Cell Longev. 2019 Apr 8;2019:6051262. doi: 10.1155/2019/6051262 (PMC6476072; doi:10.1155/2019/6051262)
Supplement: Supplementary Materials — Supplementary Figure 1: Oil Red staining identified lipid droplets in the FFA (0.4 mM OA + 0.2 mM palmitate) group. Supplementary Table 1: sequences of siRNA. Supplementary Table 2: primer sequences for qRT-PCR used in the study. [file 6051262.f1.docx]

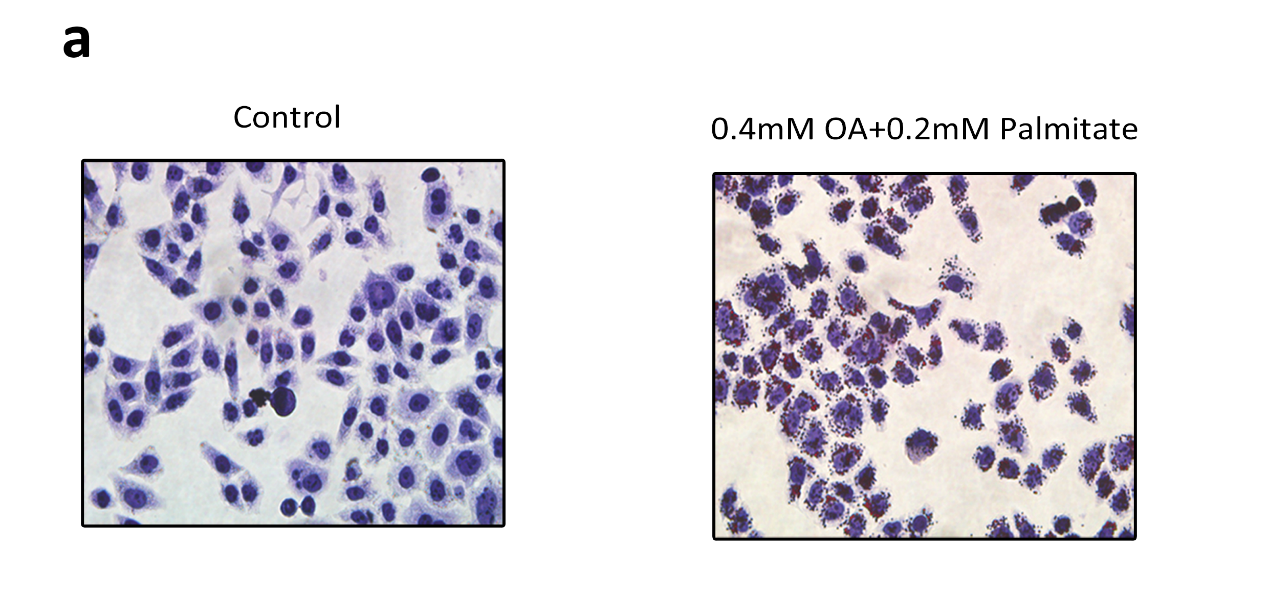


**Supplementary figure 1.** oil red staining identified lipid droplets in the FFA (0.4 mM OA+0.2 mM palmitate) group

| Target | Sequence |
| --- | --- |
| siRNA-Higd1a | 5’- AGUCACUAGUAAUUCGGUC -3’ |
| siRNA-HIF-1a | 5’- AAGTCTGCAACATGGAAGGTA -3’ |
| siRNA-PGC-1a | 5’- AAGATGACAGACCTCAGGCAG-3’ |
| SiRNA-NC | 5’-ACGUGACACGUUCGGAGAATT-3’ |
| shRNA-Higd1a | 5’-GAGCAGAGGAAATACAAAGATGTCCATTC-3’ |
| shRNA-NC | 5’-CCATTCACTTGATCCACATGCGTGTAGCA-3’ |

**Supplementary Table 1. Sequences of siRNA and shRNA**

**Supplementary Table 2. Primer sequences for qRT-PCR in study.**

| Target | Sequence |
| --- | --- |
| Higd1a | Sense: 5’- AAGAGGCACCATTCGTACCC -3’ |
|  | Anti-sense: 5’- ACCAACAGTCATTGCTCCTACA -3’ |
| HIF-1a | Sense: 5’- GAACGTCGAAAAGAAAAGTCTCG -3’ |
|  | Anti-sense: 5’- CCTTATCAAGATGCGAACTCACA -3’ |
| PGC-1a | Sense: 5’-TGAAGACGGATTGCCCTCATT -3’ |
|  | Anti-sense: 5’-GCTGGTGCCAGTAAGAGCTT-3’ |
| GAPDH | Sense: 5’- GGAGCGAGATCCCTCCAAAAT -3’ |
|  | Anti-sense: 5’- GGCTGTTGTCATACTTCTCATGG −3’ |
